# miR-141 exacerbates lung ischemia-reperfusion injury by targeting EGFR/β-catenin axis-mediated autophagy

**DOI:** 10.18632/aging.204137

**Published:** 2022-08-16

**Authors:** Miao Yang, Xiaomei Ling, Jinfang Xiao

**Affiliations:** 1 Department of Anesthesiology, Nanfang Hospital, Southern Medical University, Guangzhou, P.R. China; 2 Department of Anesthesiology, Guizhou Province People’s Hospital, Guiyang, P.R. China

**Keywords:** microRNA-141 (miR-141), EGFR, β-catenin, lung ischemia reperfusion injury, autophagy

## Abstract

Some microRNAs (miRNAs) play important roles in lung ischemia-reperfusion injury (LIRI) injury. Here, this study aimed to examine whether miR-141 was related to lung ischemia-reperfusion injury (IRI) via regulating autophagy and the epidermal growth factor receptor (EGFR), and to explore the underlying signal transduction pathways. To this end, we constructed the LIRI cell model and mouse models, separately. According to RT-qPCR and Western blotting (WB) analysis results, miR-141 up-regulation together with β-catenin and EGFR down-regulation within mouse pulmonary microvascular endothelial cells (PMVECs) or lung tissues was related to lung IRI. Besides, we conducted dual-luciferase reporter assay, which suggested the binding of EGFR to miR-141. In addition, we carried out TUNEL staining, HE staining, and flow cytometric analysis to assess the apoptosis of PMVECs and the injury to mouse lung tissues. Furthermore, we performed light-chain immunofluorescence assay to examine autophagosomes within PMVECs. According to our results, miR-141 suppressed β-catenin level through reducing EGFR level. Besides, the miR-141/EGFR/β-catenin axis enhanced autophagy to aggravate LIRI. To sum up, miR-141 suppresses EGFR expression to inhibit β-catenin level, which subsequently aggravates autophagy and complicates LIRI. The above results offer the candidate therapeutic target for the treatment of lung IRI.

## INTRODUCTION

Lung transplantation has become the only effective treatment for end-stage benign lung diseases induced by various causes [1]. However, compared with other organ transplantation (such as heart transplantation, kidney transplantation, and liver transplantation), the 5-year survival rate after lung transplantation is reported to be about 60%, and about 24% of patients die while waiting for donor lungs [2]. There are many reasons responsible for the failure or low long-term survival rate of lung transplantation, among which, lung ischemia-reperfusion injury (LIRI) is the main cause. Ischemia-reperfusion injury (IRI) is a common and important pathophysiological process during lung transplantation, which mainly includes local ischemic injury and reperfusion injury [3]. In addition, LIRI may be related to rejection after surgery. However, the mechanism of LIRI remains unclear at present. Therefore, it is of great significance to examine the precise pathogenesis of LIRI for alleviating LIRI and improving lung transplantation outcome.

MicroRNAs (miRNAs) are the small endogenous non-coding RNAs that contain approximately 18-25 nucleotides, which function to modulate gene expression at translational and transcriptional levels. MiRNAs can specifically recognize and bind to the target mRNAs by forming the RNA-induced silencing complex (RISC), thus regulating the expression of specific genes [4]. In addition, miRNAs are suggested to exert vital parts in the modulation of diverse pathophysiological processes within mammals, and more than 50% of mammalian protein-coding genes are regulated by miRNAs. MiRNAs have been verified in many reports to participate in IRI, which mainly regulate cell proliferation, differentiation, apoptosis, autophagy, inflammation and immune response. Besides, there are many studies on the roles of miRNAs in in kidney, liver, brain and heart IRI [5–9]. Typically, miR-141 shows low expression in PC12 and H9C2 cells experiencing IRI, and it also aggravates IRI. We are interested in exploring the function and underlying mechanism by which miR-141 regulates LIRI [10, 11]. According to our bioinformatics analysis results at TargetScan website, miR-141 potentially targeted and suppressed the expression of epidermal growth factor receptor (EGFR). EGFR has been previously suggested to exert a vital part in cell growth, differentiation, autophagy and apoptosis, and it is also involved in some signaling pathways [12, 13]. Meanwhile, β-catenin is reported to be closely related to EGFR. Under the action of EGFR, β-catenin accumulates in the nucleus to induce cell proliferation; on the other hand, β-catenin can directly target EGFR and activate the downstream signaling pathways [14]. Moreover, the β-catenin signaling pathway possibly exerts a vital part in resisting IRI. Based on the above observations, the miR-141/EGFR/β-catenin axis is suggested to participate in LIRI occurrence [15]. In this study, we reported the damage of miR-141 against IRI in a mouse model and discovered that the inhibition of β-catenin signaling induced by EGFR constituted a critical mechanism.

## RESULTS

### miR-141 levels increased within PMVECs and lung tissues experiencing IRI

**Figure 1:**
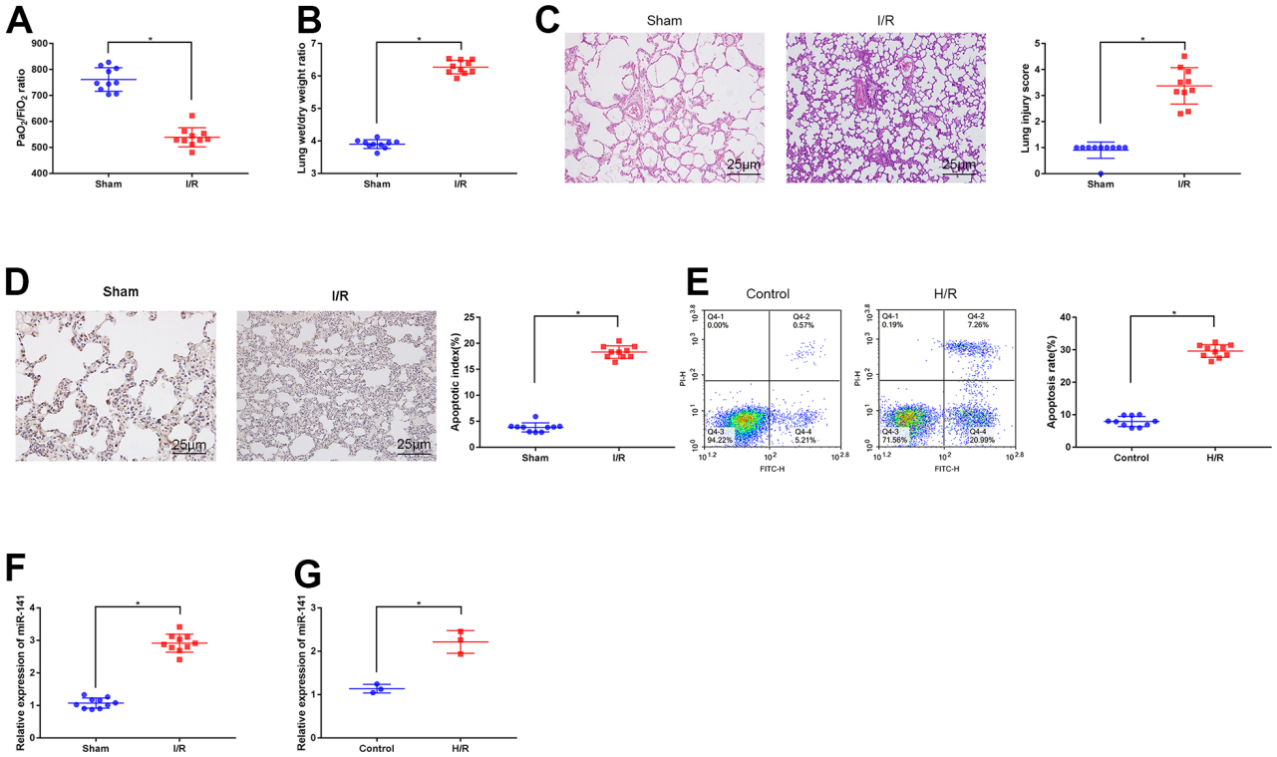
**Expression of miR-141 upregulates in lung tissues and PMVECs of I/R injury.** (**A**) Blood gas analyzer was used to detect the blood gas in arterial blood in left ventricle of mice. (**B**) The statistical graph of W/D ratio of lung tissues of mice. (**C**) HE staining results of lung tissues (× 400) and lung injury scores. TUNEL staining (**D**) and flow cytometry assay (**E**) was used to detect the apoptosis of mouse lung cells (× 200) and PMVECs. The expression of miR-141 in mouse lung tissues (**F**) and PMVECs (**G**) were determined by RT-qPCR. * *p* < 0.05 was considered statistically significant. The experiment was repeated three times independently. Results were expressed as the mean ± SD.

Firstly, I/R surgery was performed to construct the lung IRI mouse models, later, a blood gas analyzer was utilized for arterial blood gas analysis in mouse left ventricle. Then, we weighed lung tissues from mice on the electronic balance to determine W/D ratio, thus evaluating edema (Figure 1A, 1B). As a result, relative to sham operation group, I/R modeling mice had reduced PaO2/FiO2 ratio within left ventricle, whereas elevated lung tissue W/D ratio. As revealed by HE staining (Figure 1C) together with TUNEL staining (Figure 1D), relative to sham operation group, I/R modeling group had aggravated apoptosis and lung tissue injury, thus confirming that LIRI mouse models were successfully constructed. Later, we used mouse PMVECs to induce H/R models to conduct *in vitro* analysis. As revealed by flow cytometric analysis (Figure 1E), H/R-challenged cells showed increased apoptosis compared with the untreated cells, suggesting that H/R cell models were successfully induced. At last, we adopted RT-qPCR to measure miR-141 levels within I/R mouse lung tissues (Figure 1F) as well as H/R cells (Figure 1G). As a result, relative to controls, miR-141 levels within I/R lung tissues and H/R cells were up-regulated, thus confirming the high expression of miR-141 within LIRI cells and tissues.

### miR-141 knockdown suppressed autophagy to mitigate LIRI *in vitro* and *in vivo*

**Figure 2:**
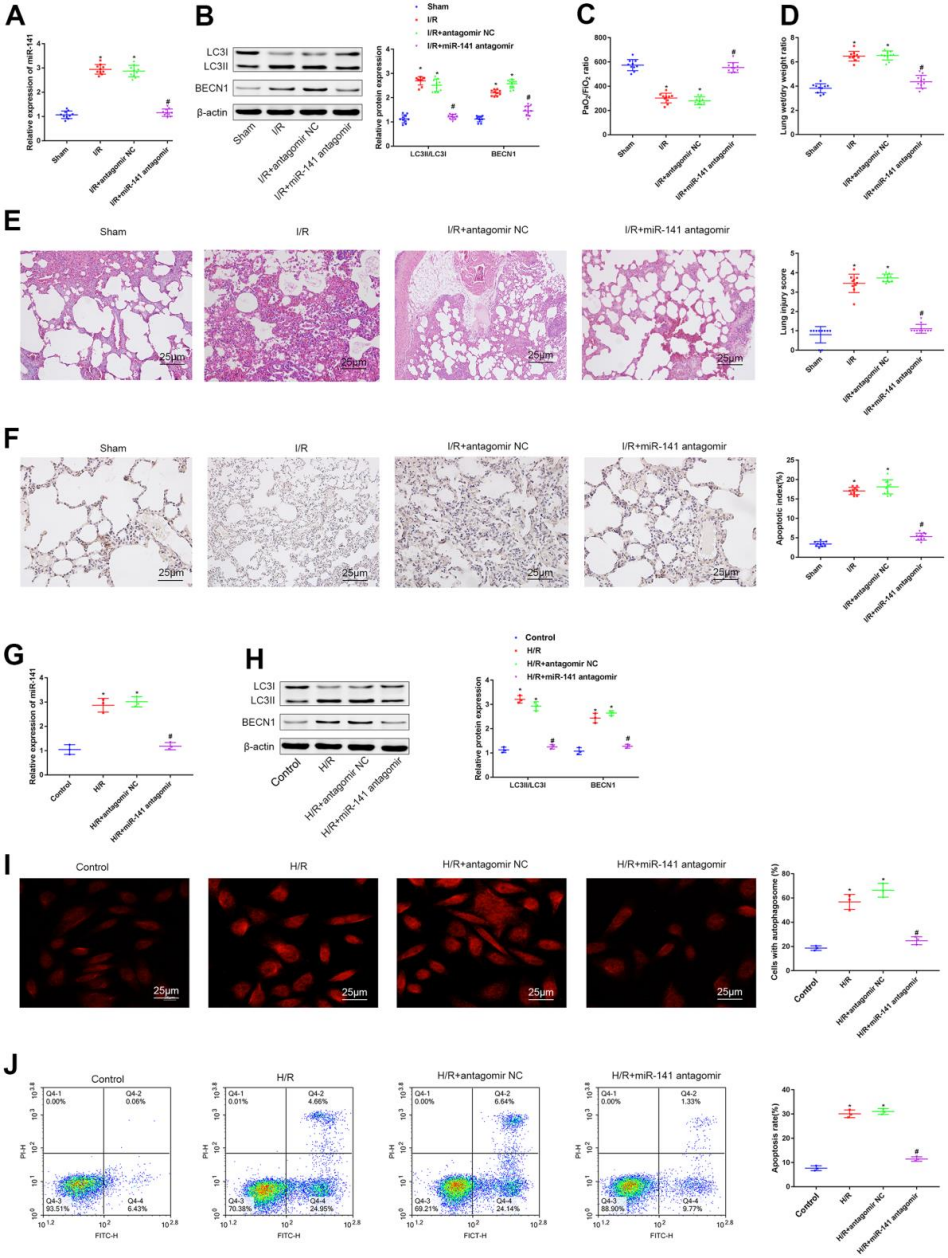
**miR-141 inhibition suppresses autophagy and relieves lung I/R injury *in vivo* and *in vitro*.** (**A**) RT-qPCR analysis for analyzing miR-141 levels within lung tissues of IRI mice. (**B**) Western blotting was used to detect the protein expression of LC3II/I and BECN1 in mouse lung tissues. (**C**) Blood gas analyzer was used to detect the blood gas in arterial blood in left ventricle of mice. (**D**) The statistical graph of W/D ratio of lung tissues of mice. (**E**) HE staining results of lung tissues (× 400) and lung injury scores. (**F**) TUNEL staining (× 200) was used to detect the apoptosis of mouse lung cells. (**G**) RT-qPCR analysis for analyzing the miR-141 expression in mouse PMVECs. (**H**) Western blotting was used to detect the protein expression of LC3II/I and BECN1 in mouse PMVECs. (**I**) LC3 immunofluorescence assay was used to detect autophagosomes. (**J**) flow cytometry assay was used to detect the apoptosis of PMVECs. * *p *< 0.05 was considered statistically significant. The experiment was repeated three times independently. Results were expressed as the mean ± SD.

As shown above, miR-141 was up-regulated within LIRI cells and tissues. For understanding the function of miR-141 in autophagy during LIRI, we divided animals into sham operation group, miR-141 antagomir or antagomir NC transfection group, and I/R modeling group. As for PMVECs, they were treated with or without miR-141 antagomir or antagomir NC and later subjected to H/R treatment. Thereafter, we performed RT-qPCR analysis for analyzing miR-141 levels within lung tissues of IRI mice (Figure 2A). As a result, compared with sham operation group, miR-141 expression increased within lung tissues from IRI modeling group and IRI modeling + antagomir NC transfection group, but that decreased after miR-141 antagomir transfection. Thereafter, we measured the autophagy-related genes (ARGs, including BECN1 and LC3II/I) levels within lung tissues through WB assay (Figure 2B). As a result, relative to sham operation group, BECN1 expression and LC3II/I ratio elevated within lung tissues from IRI modeling group and IRI modeling+antagomir NC transfection group. Relative to I/R modeling+antagomir NC transfection group, BECN1 expression and LC3II/I ratio decreased after miR-141 antagomir transfection. Arterial blood gas analysis was conducted in mouse left ventricle by using the blood gas analyzer, then, edema was assessed through calculating W/D ratio (Figure 2C, 2D). As a result, relative to sham operation group, I/R modeling group and I/R modeling+antagomir NC transfection group had reduced PaO2/FiO2 ratio, whereas elevated lung tissue W/D ratio (p<0.05). Besides, I/R modeling+ miR-141 antagomir transfection mice showed increased PaO2/FiO2 ratio whereas decreased W/D ratio compared with I/R modeling mice receiving antagomir NC transfection (p<0.05). As suggested by HE staining (Figure 2E) as well as TUNEL staining (Figure 2F) results, relative to sham operation group, I/R modeling group and I/R modeling+antagomir NC transfection group had increased apoptosis and aggravated lung tissue injury, while miR-141 antagomir transfection mitigated cell injury and inhibited apoptosis. Consistent results were obtained from each cell experiment after transfection (Figure 2G–2J). As suggested by the above findings, miR-141 knockdown mitigated LIRI and inhibited autophagy *in vitro* and *in vivo*.

### miR-141 inhibited β-catenin level by the negative regulation of EGFR level

**Figure 3:**
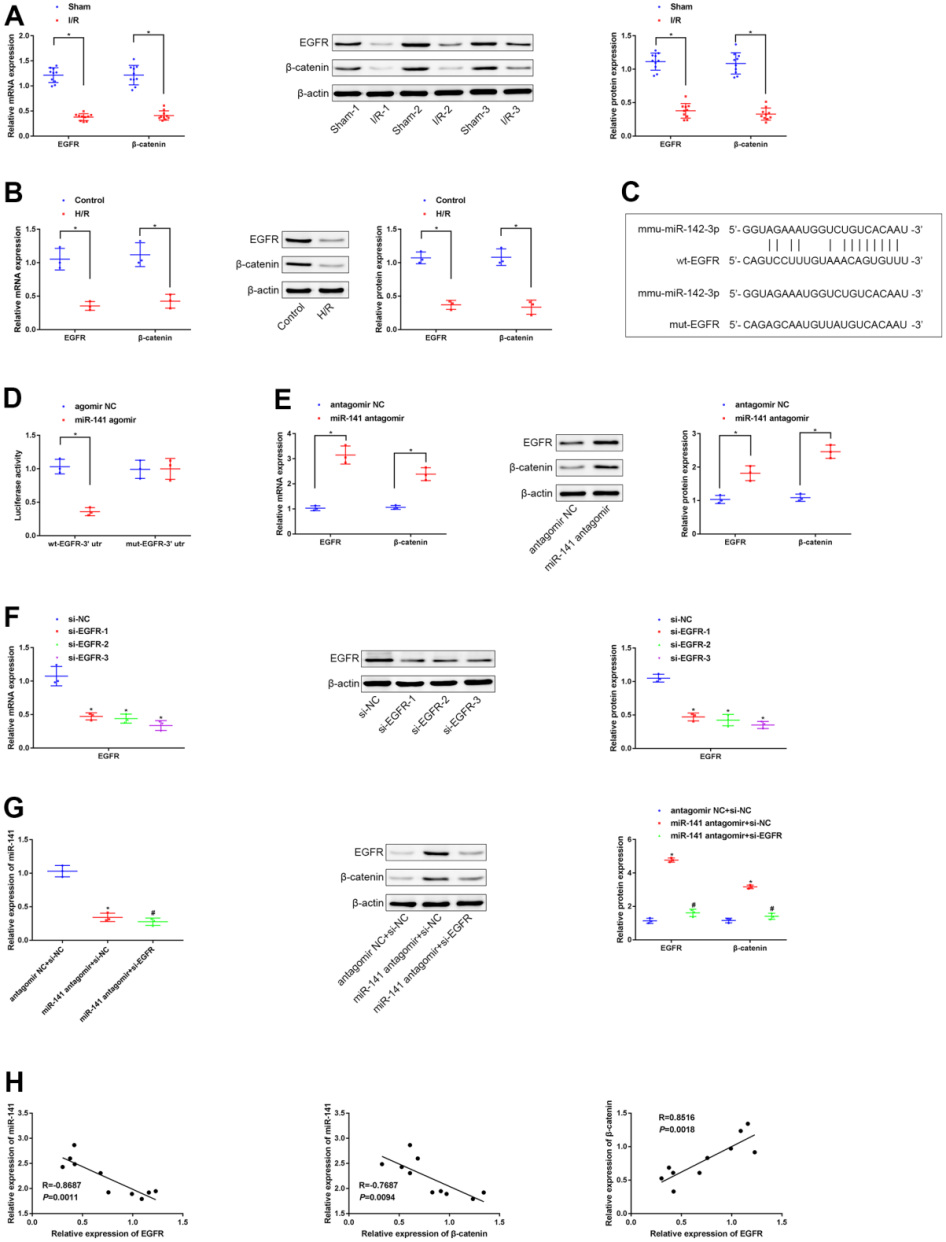
**miR-141 represses EGFR to suppress expression of β-catenin.** (**A**, **B**) RT-qPCR and Western blot assays were used to detect the expression of EGFR and β-catenin in lung tissues of mice and PMVECs with I/R injury. (**C**) the predicted binding sites between miR-141 and EGFR based on TargetScan database. (**D**) the dual luciferase reporter gene assay proved that validation of the binding relationship between miR-141 and EGFR. (**E**) RT-qPCR and Western blot assays were used to detect the expression of EGFR and β-catenin in response to silencing miR-141 in H/R cell model. (**F**) RT-qPCR assay was used to detect the silencing efficiency of si-EGFR-1/-2/-3 in H/R-exposed mouse PMVECs. (**G**) The expression of miR-141, EGFR and β-catenin in PMVECs by RT-qPCR and Western blot analysis. (**H**) Pearson correlation analysis of the correlation between miR-141 and EGFR, between miR-141 and β-catenin, as well as between EGFR and β-catenin. * *p* < 0.05 was considered statistically significant. The experiment was repeated three times independently. Results were expressed as the mean ± SD.

As predicted by the TargetScan database, miR-141 potentially targets and suppresses the EGFR level in mice. As a result, this study speculated that miR-141 targeted the EGFR level and suppressed β-catenin level in mice, and such speculation was further validated. According to RT-qPCR and WB results, the expression of β-catenin and EGFR decreased within LIRI cells and tissues relative to that within control cells and non-carcinoma tissues (Figure 3A, 3B). Moreover, analysis based on microarray suggested that there was a binding site in miR-141 for EGFR (Figure 3C). We conducted dual luciferase reporter gene assay to examine such targeting association of miR-141 with EGFR by using the mouse PMVECs (Figure 3D). As a result, the fluorescence intensity in PMVECs exposed to H/R and co-transfection with wt-EGFR-3’UTR and miR-141 agomir was markedly reduced in comparison with that in cells exposed to H/R and agomir NC transfection, and that in cells exposed to H/R and co-transfected with mut-EGFR-3’UTR and miR-141 agomir was not significantly different. We further conducted RT-qPCR and WB assays to detect the role of miR-141 knockdown in β-catenin and EGFR expression in H/R cell model (Figure 3E). According to our findings, miR-141 showed negative regulation on β-catenin and EGFR expression. Later, the cell line showing the highest silencing efficiency (si-EGFR-3) was screened to conduct later analyses (Figure 3F). Later, miR-141 antagomir + si-EGFR, miR-141 antagomir + si-NC, or antagomir NC + si-NC were co-transfected into H/R-challenged PMVECs. The expression levels of miR-141, β-catenin and EGFR were measured by RT-qPCR and WB assays (Figure 3G). As a result, miR-141 antagomir increased β-catenin expression within H/R-challenged PMVECs, while si-EGFR transfection abolished such effect. At last, associations of miR-141 with EGFR level, miR-141 with β-catenin level, and EGFR with β-catenin level within lung tissues of 10 LIRI mice were examined through Pearson correlation analysis (Figure 3H). As a result, miR-141 level was inversely related to EGFR and β-catenin levels, whereas EGFR level was positively correlated with β-catenin level. As a result, miR-141 suppressed β-catenin level by targeting and inhibiting EGFR.

### The miR-141/EGFR/β-catenin axis enhanced autophagy and aggravated H/R-mediated injury of mouse PMVECs

**Figure 4:**
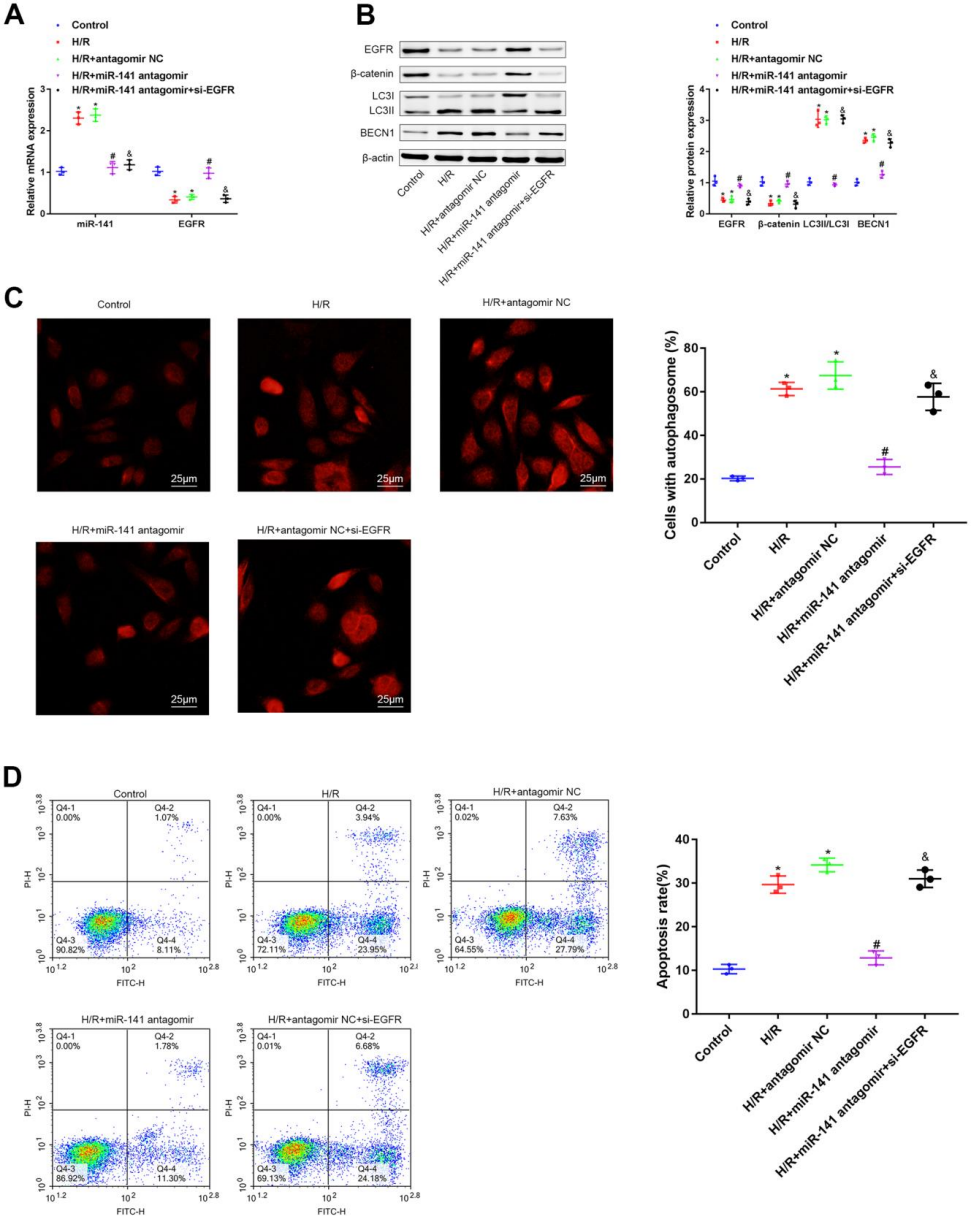
**miR-141/EFGR/β-catenin axis gives rise to autophagy, contributing to the progression of H/R-induced injury *in vitro*.** (**A**) miR-141 and EGFR expression in mouse PMVECs was measured by RT-qPCR. (**B**) EGFR, β-catenin, LC3II/I and BECN1 expression in mouse PMVECs were measured by Western blot analysis. (**C**) LC3 immunofluorescence assay (× 400) was used to detect autophagosomes of PMVECs. (**D**) the apoptosis rate of PMVECs was measured by flow cytometry assay. * *p* < 0.05 was considered statistically significant. The experiment was repeated three times independently. Results were expressed as the mean ± SD.

For investigating the function of miR-141/EGFR/β-catenin axis in autophagy during the H/R-mediated injury, we transfected PMVECs with or without miR-141 antagomir + si-EGFR, miR-141 antagomir, or antagomir NC, and later treated cells under H/R conditions. Firstly, EGFR and miR-141 expression levels were determined through RT-qPCR assay (Figure 4A). As a result, relative to non-H/R-exposed PMVECs, miR-141 level was up-regulated, whereas EGFR level was down-regulated in cells treated under H/R environment and in cells treated with H/R and antagomir NC simultaneously. Relative to mouse PMVECs subjected to H/R and antagomir NC treatment, miR-141 level decreased within mouse PMVECs subjected to H/R exposure combined with miR-141 antagomir+si-EGFR or miR-141 antagomir transfection, whereas EGFR level increased within mouse PMVECs exposed to H/R and miR-141 antagomir transfection. Relative to mouse PMVECs exposed to H/R and miR-141 antagomir transfection, EGFR level declined after transfection with miR-141 antagomir + si-EGFR. According to WB assay (Figure 4B), LC3 immunofluorescence analysis (Figure 4C) as well as flow cytometric analysis (Figure 4D), relative to non-H/R-exposed mouse PMVECs, β-catenin and EGFR levels declined within cells subjected to H/R exposure and cells subjected to H/R exposure plus antagomir NC transfection, while BECN1 level, LC3II/LC3I, apoptosis rate and autophagosomes increased. Compared with PMVECs subjected to H/R exposure and antagomir NC transfection, β-catenin and EGFR levels increased in cells exposed to H/R and miR-141 antagomir transfection, whereas BECN1 level, LC3II/LC3I, apoptosis rate and autophagosomes decreased. But further si-EGFR exposure had contrary findings. As a result, the miR-141/EGFR/β-catenin axis deteriorated injury to cells under H/R exposure through promoting autophagy.

### miR-141/EGFR/β-catenin axis promoted autophagy *in vivo* to deteriorate LIRI

**Figure 5:**
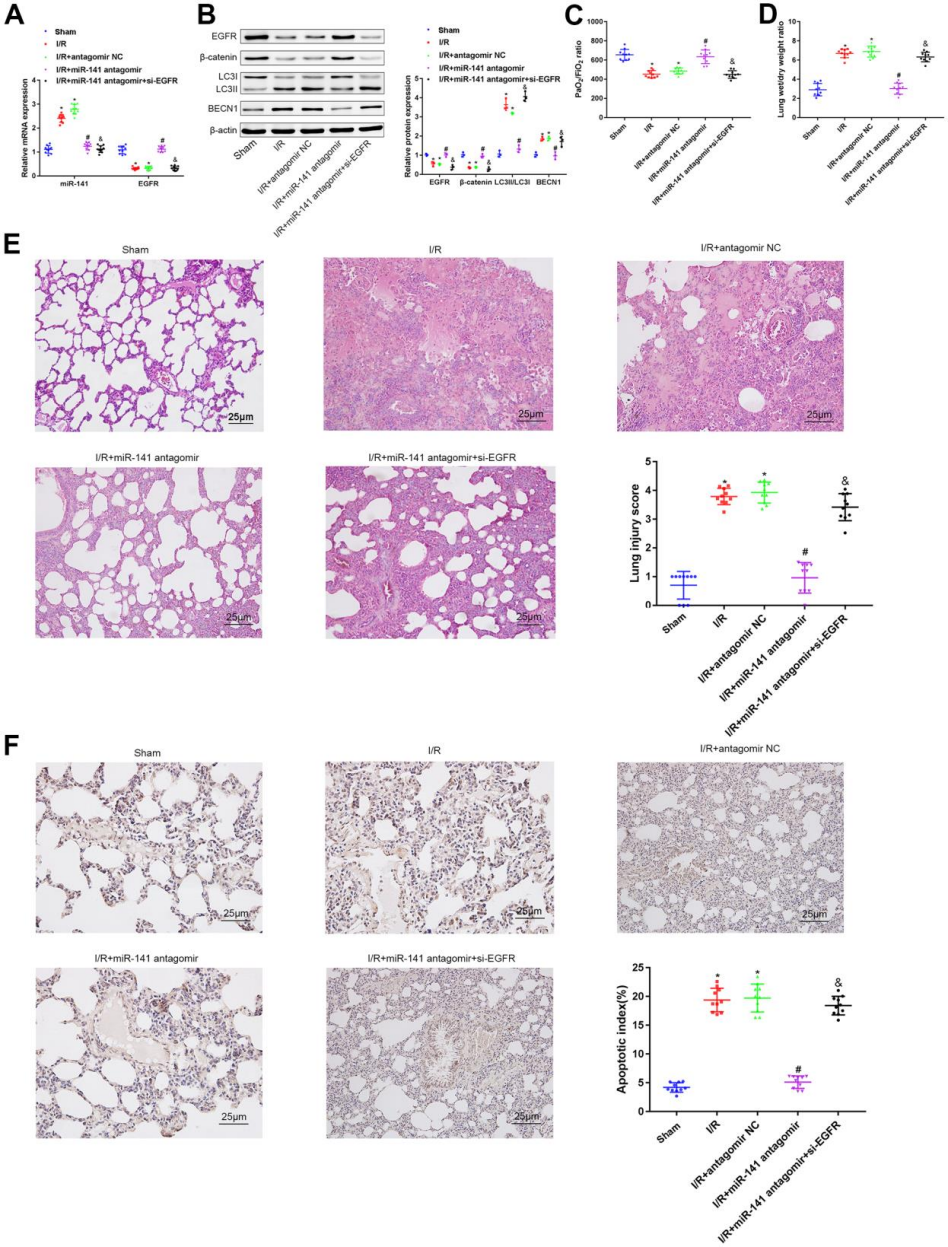
**miR-141 inhibits EGFR to downregulate β-catenin, thereby enhancing autophagy to promote lung I/R injury *in vivo*.** (**A**) RT-qPCR was used to detect the expression of miR-141 and EGFR in mouse lung tissues. (**B**) Western blotting was used to detect the protein expression of ERFG, β-catenin, LC3II/I and BECN1 in mouse lung tissues. (**C**) Blood gas analyzer was used to detect the blood gas in arterial blood in left ventricle of mice. (**D**) The statistical graph of W/D ratio of lung tissues of mice. (**E**) HE staining results of lung tissues (× 400) and lung injury scores. (**F**) TUNEL staining (× 200) was used to detect the apoptosis of mouse lung cells. * *p* < 0.05 was considered statistically significant. The experiment was repeated three times independently. Results were expressed as the mean ± SD.

For investigating the role of miR-141/EGFR/β-catenin axis in autophagy during LIRI *in vivo*, we classified mice into sham operation group, miR-141 antagomir transfection group, antagomir NC transfection group, and miR-141 antagomir + si-EGFR transfection group; thereafter, all mice were treated under I/R environment. Firstly, EGFR and miR-141 levels within mouse lung tissues were measured through RT-qPCR (Figure 5A). As a result, relative to sham operation group, miR-141 level elevated in I/R-challenged mice as well as in I/R-challenged mice under antagomir NC transfection, whereas EGFR level reduced. Relative to I/R-challenged mice under antagomir NC transfection, miR-141 level decreased in I/R-challenged mice under miR-141 antagomir transfection and those under miR-141 antagomir + si-EGFR transfection, and EGFR level increased in I/R-challenged mice under miR-141 antagomir transfection. Relative to I/R-challenged mice under miR-141 antagomir transfection, EGFR level reduced in I/R-challenged mice under miR-141 antagomir+si-EGFR transfection. As shown by WB assay (Figure 5B), arterial blood gas analysis (Figure 5C), W/D ratio measurement (Figure 5D), HE staining (Figure 5E) together with TUNEL staining (Figure 5F) results, relative to sham operation group, β-catenin and EGFR levels and PaO2/FiO2 ratio decreased, whereas LC3II/LC3I ratio, W/D ratio, BECN1 level, apoptosis and lung tissue injury increased in I/R-challenged mice and in I/R-challenged mice under antagomir NC transfection. Relative to I/R-challenged mice under antagomir NC transfection, β-catenin and EGFR levels, together with PaO2/FiO2 ratio elevated, but LC3II/LC3I ratio, W/D ratio, BECN1 level, apoptosis and lung tissue injury decreased in I/R-challenged mice under miR-141 antagomir transfection. Besides, miR-141 antagomir combined with si-EGFR treatment led to contrary results compared with those obtained by miR-141 antagomir treatment alone. In conclusion, miR-141 targeted and suppressed EGFR level in LIRI tissues to suppress β-catenin level and suppressing β-catenin aggravated autophagy for aggravating LIRI *in vivo*.

## DISCUSSION

Ischemia and reperfusion after ischemia often occur in a variety of clinical processes, such as major surgery and organ transplantation, which are usually accompanied with serious destructive consequences, resulting in tissue damage [16]. Undoubtedly, restoring the perfusion of ischemic tissue is necessary to maintain the physiological function of the tissue. However, reperfusion itself can also trigger a series of complex events, which is the so-called ischemia-reperfusion injury (IRI). Take lung transplantation as an example, in the process of lung transplantation, although complete and long-term hypoxia lasts for only several hours, it causes unimaginable but unavoidable consequences [1]. Severe IRI leads to primary graft dysfunction (PGD), which accounts for the main cause of high short-term and long-term morbidity and mortality rates after lung transplantation [17]. At present, no specific treatment is available for the prevention of IRI. To offer new therapeutic targets for treating LIRI, the present work examined the role of miR-141 in LIRI and its associations with β-catenin and EGFR. It is reported that I/R treatment induces the low expression of miR-141 in H9C2 and PC12 cells [10, 11]. miR-141 inhibitors significantly aggravate IRI by promoting cell apoptosis. There are many studies on the role of apoptosis in IRI. It is found that inhibition of apoptosis can reduce IRI [18–20]. Apoptosis is closely related to autophagy that plays dual roles (both positive and negative) in apoptosis [21]. For example, midazolam has been reported to induce autophagy of lung cancer A549 cells, and inhibition of autophagy mitigates the cytotoxicity of receptor tyrosine kinase inhibitors [22], indicating that autophagy has different functions in a variety of tissue cells. It should also be noted that, autophagy is a double-edged sword. A certain degree of autophagy represents the self-protective mechanism of cell self-renewal, which contributes to adapting to adverse environment and improving cell tolerance, but excessive autophagy will lead to cell death [23]. Studies have shown that I/R leads to excessive autophagy, which aggravates damage to the liver, kidney, heart, and lung [2, 7, 9, 10, 16, 18]. As suggested by our results, miR-141 showed low expression within lung tissues in the IRI mouse model. Moreover, findings in this study indicated that miR-141 knockdown within PMVECs inhibited autophagy and apoptosis, thus ameliorating LIRI, which was related to the up-regulation of EGFR and β-catenin expression.

The change in cellular behavior is closely related to protein expression. LC3II/I ratio has been extensively used as a biomarker for autophagy, which indicates the autophagy of esophageal cancer cells. On the other hand, Beclin1 exerts an important part in the formation of autophagosomes and the fusion of autophagosomes with lysosomes. EGFR is a key factor that regulates cell proliferation [24], which participates in multiple signaling pathways and induces cell proliferation, differentiation and invasion. Some studies have shown that EGFR is significantly correlated with the autophagy activity of lung cancer cells [25]. The downregulation of EGFR inhibits β-catenin expression in the nucleus and promotes apoptosis; at the same time, the downregulation of β-catenin weakens the targeted activation of EGFR and accelerates apoptosis [26]. In the present study, down-regulation of miR-141 decreased LC3II/I ratio and suppressed BECN1 expression, thus leading to LIRI-induced autophagy. Meanwhile, the expression of both EGFR and β-catenin in PMVECs decreased by H/R treatment. Further, miR-141 targeted EGFR to inhibit the expression of β-catenin. Therefore, miR-141 was confirmed to have an important function in enhancing LIRI through specifically inhibiting EGFR by suppressing β-catenin.

In addition, results from HE staining suggested interstitial and perivascular pulmonary edema, interalveolar bleeding, alveolar tubular “hyaline” changes, and neutrophil/macrophage/polymorphonuclear cell infiltration into alveolar cavity and pulmonary interstitium post-LIRI, and these were mitigated by inhibiting miR-141 expression. LIRI was related to the severity of inflammation and neutrophil infiltration. Meanwhile, miR-141 has been suggested to exert a vital part in the regulation of immune cells during the inflammatory response via the HMGB1 gene and protein pathway [27]. In response to IL-36, miR-141-5p expression increased within HTR-8/SVneo and PTC cells [28]. Thus, our future studies will focus on the role of miR-141/EGFR/β-catenin axis in inflammatory response caused by IRI.

In conclusion, inhibition of miR-141 reduces the IRI-induced autophagy within lung tissues, which is achieved through suppressing EGFR to restrain β-catenin, thereby inhibiting LIRI occurrence. Such results help to further understand the LIRI mechanism and the role of miR-141 as a therapeutic target for the clinical LIRI treatments.

## MATERIALS AND METHODS

### Establishment of the LIRI mouse model

Altogether 68 6-8-week-old C57BL/6J male mice weighing 20-26g were obtained and raised under the light/dark cycle of 12-h/12 h, and they were allowed to eat food freely. Later, all animals were classified as sham-operation (n = 10), ischemia alone (n = 10), and I/R (n = 48) groups. The 3% pentobarbital sodium was injected into mice intraperitoneally at a dose of 50 mg/kg for anesthesia, followed by intubation with the 20G needle through tracheotomy and connection to the rodent ventilator with controlled volume (Harvard Inspira ASV). Then, we opened the 3^rd^-4^th^ lateral intercostal space in left thoracic cavity and mice were given intraperitoneal injection of heparin (100 U/kg, Qianhong, Changzhou, China). At 5 min later, we used a non-invasive microvascular clip to clamp the left pulmonary hilum for 1 h, then we removed the clip for 2 h of reperfusion in mice. Mice in sham operation group (n = 10) received identical procedure except for pulmonary hilum clamping. After reperfusion for 1 h, we drew arterial blood from the left ventricle. Thereafter, excessive sodium pentobarbital (150 mg/kg) was injected into mice intraperitoneally for euthanasia, followed by resection of the left lung. At 48 h prior to modeling, mice in I/R modeling group (n = 12) were administered with or without miR-141 antagomir, miR-141 antagomir + siRNA targeting EGFR (si-EGFR, Shanghai GenePharma Co., Ltd., Shanghai, China) or antagomir negative control (NC) through the trachea. In brief, we diluted si-EGFR to 20 μmol/L and then exposed mouse glottis directly under the animal laryngoscope. Later, we inserted the nebulizer into mouse trachea via the mouth to nebulize si-EGFR (100 μL) to mice. Thereafter, we conducted ischemia for 1 h and reperfusion for 2 h. As for mice in I/R + miR-141 antagomir + si-EGFR group, they were exposed to 2 h of miR-141 antagomir treatment, Followed by si-EGFR treatment. Later, we chose 10 successful mouse models to conduct subsequent experiments.

### Establishment of the *in vitro* cell model of hypoxic reoxygenation (H/R)

Pulmonary microvascular endothelial cells (PMVECs) were obtained from Cell Biologics Inc. (Chicago, IL, USA), cultivated within the M-1168 medium (Cell Biologics), and incubated within the traditional incubator under 37° C and 5% CO_2_ conditions. During the culture process, we adjusted cell density based on the cell growth rate, and then we grew cells into the 6-well plates. After reaching 80-90% confluency, Lipofectamine 2000 (Invitrogen, Carlsbad, CA, USA) was utilized to transfect cells with miR-141 antagomir, agomir NC, miR-141 antagomir + si-NC, antagomir NC + si-NC, or miR-141 antagomir + si-EGFR. Prior to transfection, Lipofectamine 2000 was blended with the transfectants, followed by dilution with medium and 5 min of standing under ambient temperature. Afterwards, 400 μL solution was added to every well and mixed sufficiently. At 6 h after cell transfection, we replaced the original medium by the freshly prepared medium and cultured the cells for another 24 h. Later, all cells were incubated within the hypoxic incubator that contained 5% CO_2_ and 95% N_2_ for a period of 12 h, followed by another 4 h of incubation within the traditional incubator for constructing the H/R cell models. Afterwards, we harvested cells to conduct further analysis. Each mouse was given humanistic treatment following the guidelines for the Care and Use of Laboratory Animals released by the National Institutes of Health (NIH). The Experimental Animal Ethics Committee approved our experimental protocols. Each experiment was conducted in strict accordance with specific guidelines for minimizing animal sufferings, discomfort and pain.

### Reverse transcription quantitative PCR (RT-qPCR)

The RNeasy Mini Kit (Qiagen, Valencia, CA, USA) was utilized to isolate total RNA. Thereafter, the extracted mRNA was prepared to cDNA by adopting the RT kit (RR047A, Takara, Kusatsu, Shiga, Japan) through reverse transcription. Later, the miRNA First-Strand cDNA Synthesis (Tailing Reaction) kit (B532451-0020, Shanghai Sangon Biotechnology Co., Ltd., Shanghai, China), which also offered the U6 upstream primer (loading reference) and universal miRNA negative primer, was applied in synthesizing cDNA from miRNA. Afterwards, we utilized the SYBR® Premix Ex TaqTM II (Perfect Real Time) kit (DRR081, Takara) to load samples. The ABI 7500 system (Applied Biosystems, Foster City, CA, USA) was employed for RT-qPCR analysis. Moreover, all the remaining primers were obtained from Shanghai Sangon Biotechnology Co., Ltd., and β-actin was used to be the loading reference. The 2^-ΔΔCt^ approach was utilized to calculate relevant product levels. Table 1 presents the sequences of each primer used in this study.

**Table 1:** The primer sequence of RT-qPCR.

Gene	Sequence (5’-3’)	Location
miR-141	F: GGGCATCTTCCAGTGCAGT R: CAGTGCGTGTCGTGGAGT	chr6: 124717952-124717969 stem-loop Sequence: 9-26
EGFR	F: GCCATCTGGGCCAAAGATACC R: GTCTTCGCATGAATAGGCCAAT	NM_207655:2197-2217 NM_207655:2275-2297
β-catenin	F: ATGGAGCCGGACAGAAAAGC R: CTTGCCACTCAGGGAAGGA	NM_007614:327-346 NM_007614:416-434
β-actin	F: GGCTGTATTCCCCTCCATCG R: CCAGTTGGTAACAATGCCATGT	NM_007393:193-212 NM_007393:325-346
U6	F: CTCGCTTCGGCAGCACA R: AACGCTTCACGAATTTGCGT	NR_003027:4-21 NR_003027:78-98

### Western blotting (WB) assay

The radio-immunoprecipitation assay that contained the phenylmethylsulfonyl fluoride was conducted to isolate total cellular or tissue proteins. Thereafter, we determined the extracted protein content through the bicinchoninic acid (BCA) protein detection kit. After dissolving the proteins (50 μg) into the 2 × SDS loading buffer, we heated the mixture for 5 min under 100° C. Then, we separated the proteins by SDS-PAGE and transferred them onto the PVDF membranes. Later, 5% skim milk powder was used to block the membranes for 1 h under ambient temperature, followed by incubation using primary antibodies (Abcam Inc., Cambridge, UK) under 4° C overnight, including β-catenin (1:2000), EGFR (1: 800), recombinant Human Beclin 1 (BECN1; 1:2000), light chain 3B (LC3B) (1:1000), together with β-actin (ab8227, 1:1000). Thereafter, we further incubated the membranes using the HRP-conjugated goat anti-rabbit secondary IgG antibody (H&L 1:2000, Abcam) for additional 1 h. Subsequently, the enhanced chemiluminescence fluorescence detection kit (Ameshame, Little Chalfont, UK) was utilized to visualize each membrane. At the same time, we employed the Bio-Rad Imaging Analysis System (Bio-Rad, Hercules, CA, USA) to obtain images, and adopted Quantity One v4.6.2 to analyze results. The gray value ratio of related protein band to β-actin (loading reference) was calculated as the relative protein level.

### Arterial blood gas analysis

Upon the completion of reperfusion, we collected arterial blood (0.4 mL) from the left ventricle by using the heparinized empty needle (1 mL). Thereafter, the blood gas analyzer (Mindray, Shenzhen, China) was utilized to analyze PaO2/FiO2 at once.

### Ratio of wet weight to dry weight (W/D)

The OHAUS-Precision electronic balance was used to weigh approximately 1/3 left lung tissues, which was denoted as the wet weight. Thereafter, we dried the tissues within an oven for 72 h under 60° C till the weight remained unchanged, which was denoted as the dry weight. Thereafter, we calculated W/D ratio to determine lung tissue edema.

### Hematoxylin-eosin (HE) staining

The 4% formaldehyde was utilized to fix lung tissues for a period of 24 h, followed by paraffin embedding, slicing and HE staining in succession. Thereafter, two investigators independently analyzed the histology under the microscope according to the scoring standard of lung injury. The scores given by them were later averaged to obtain the eventual score. To be specific, the score of 0 indicated no injury, while that of 4 stood for severe injury, which included bleeding, alveolar congestion, alveolar wall thickness, neutrophil counting, and interstitial edema.

### LC3 immunofluorescence examination

After 4% paraformaldehyde fixation and 0.3% Triton X-100 permeabilization, the bovine serum albumin (BSA) was used to block cells, and rabbit anti-LC3B antibody (ab51520, 1:2000, Abcam) was later used to incubate cells under 4° C overnight, followed by another 1 h of incubation by the Alexa Fluor 488-labeled donkey anti-rabbit IgG secondary antibody (A21206, 1:500, Thermo Fisher Scientific, Waltham, MA, USA) under ambient temperature. Afterwards, we utilized the confocal laser microscope (Leica TCS SP5II STED, Mannheim, Germany) to observe cells. Altogether 200 or more cells were calculated from every section to determine the LC3 spot cell (autophagosome) proportion.

### Terminal deoxynucleotidyl transferase-mediated 2’-deoxyuridine 5’-triphosphate-biotin nick end-labeling (TUNEL) staining

We conducted TUNEL staining in line with specific protocols (Merck Millipore, Billerica, MA, USA). Thereafter, investigators counted the TUNEL-positive cells, which had brown nuclei, by using the light microscope (Olympus, Tokyo, Japan) independently (5).

### Dual luciferase reporter gene assay

In this study, Shanghai GenePharma Co., Ltd was responsible for designing and synthesizing the mutant (mut) and wild-type (wt) reporter plasmids (pGL3-mut-EGFR-3’UTR and pGL3-wt-EGFR-3’UTR) for EGFR-3’UTR. Then, mouse PMVECs were co-transfected with miR-141 agomir or Agomir NC and mut-EGFR-3’UTR or wt-EGFR-3’UTR, separately. We lysed cells at 48 h after transfection. Later, the luciferase assay kit (K801-200, Biovision, Milpitas, CA, USA) was employed to measure luciferase activities through the dual luciferase reporter gene assay system (Promega, Madison, WI, USA) in line with specific protocols. Then, we determined the relative light unit (RLU) ratio of firefly luciferase to renilla luciferase to measure the target reporter gene activation level, and renilla luciferase was used to be the endogenous control.

### Flow cytometric analysis

The Annexin V-fluoresceine isothiocyanate (FITC)/propidiumiodide (PI) kit (KeyGEN Biotech., Co., Ltd., Nanjing, Jiangsu, 199 China) was utilized to measure cell apoptosis in accordance with specific instructions. Later, the flow cytometer (FACSCalibur, BD Biosciences, San Jose, CA, USA) was adopted for result analysis.

### Statistical analysis

The SPSS21.0 (IBM Corp. Armonk, NY, USA) was employed for all statistical analyses. We presented measurement data in a form of mean ±SD. For normally distributed unpaired data with variance homogeneity, we analyzed them by unpaired t-test between both groups. In addition, ANOVA along with Tukey’s post-hoc tests were adopted to compare across several groups. Also, the repeated measures ANOVA was employed to statistically analyze time-based measurements inside every group, and multiple comparisons were completed using Bonferroni’s post-hoc test. The associations across miR-141, β-catenin and EGFR were determined by the Pearson correlation coefficient. A difference of *p* < 0.05 suggested statistical significance.
